# Improving Patient Care and Streamlining Follow-Up: Compliance With National Institute for Health and Care Excellence (NICE) Guidelines for Pediatric Distal Radius Buckle Fractures

**DOI:** 10.7759/cureus.73624

**Published:** 2024-11-13

**Authors:** Faris Ali, Mohamed Elmubark, Raja Muhammad Mussab, Islam Mubark, Nithish Jayakumar, Neil Ashwood

**Affiliations:** 1 Department of Trauma and Orthopedics, Queen’s Hospital, University Hospitals of Derby and Burton NHS Foundation Trust, Burton Upon Trent, GBR; 2 Department of Trauma and Orthopedics, Queen Elizabeth Hospital, Gateshead, GBR; 3 Department of Trauma and Orthopedics, Russells Hall Hospital, Dudley Group NHS Foundation Trust, Birmingham, GBR

**Keywords:** buckle fracture, emergency department, fracture clinic, full cast, nice guidelines, torus fracture, wrist splint

## Abstract

Introduction: Torus fractures, also known as buckle fractures, are among the most common types of fractures seen in children who present to the emergency department (ED). These injuries usually occur when a child falls onto an outstretched hand, resulting in the compression and buckling of the dorsal cortex of the radius while the volar cortex remains intact. These fractures generally have a good prognosis and heal well with simple immobilization with a low risk of complications. However, current treatment practices often involve using a rigid cast and scheduling multiple follow-up clinic visits, which increases the hospital's financial strain.

Materials and methods: We conducted an initial audit that reviewed the practice in our unit between August and October 2017 at Queen's Hospital, Burton Upon Trent, United Kingdom. It included all children below the age of 16 who had radiograph images confirming distal radius buckle fractures and have been referred to the fracture clinic. Patient demographics, clinic visits, treating doctor grade/specialty, radiographs, initial and final treatment outcomes, and cast application were collected. After the initial audit, compliance with National Institute for Health and Care Excellence (NICE) guidelines was promoted through the education of healthcare providers. A second audit was performed within 12 months to reassess the compliance.

Results: This study looked at the management of pediatric distal radius buckle fractures in a cohort of 152 patients, of which 65 and 87 children were included in audit cycles I and II, respectively. In the ED, splint usage increased from 0% in the first cycle (all children initially treated in a back slab) to 20% following new recommendations. In the fracture clinic, there was a notable improvement in the use of splints over full plaster casts between the first and second cycles. Initially, in the first cycle, only 5% of patients were treated in a splint, with 95% receiving full plaster casts. Following recommendations, splint use increased significantly in the second cycle, rising to 53%, while cast use decreased to 47%. In the first audit, only 7.7% (five patients) were discharged at the first visit, compared to 44.8% (39 patients) in the second audit. In the first audit, 86.2% (56 patients) required a second visit, whereas in the second audit, this decreased to 55.2% (48 patients). Four individuals received a cast owing to splint size difficulties or patient preferences.

Conclusion: Despite the improvement seen regarding compliance with NICE guidelines, work is still needed to further enhance compliance. Staff education and optimizing splint availability will be a priority to reduce the burden on fracture clinic resources by unnecessary follow-up appointments.

## Introduction

In the United Kingdom, an estimated 500,000 distal radius torus fractures occur annually in the emergency departments (ED) [1]. Distal radius torus fracture commonly presents in children below 14 years of age due to low-energy falls. It presents when the dorsal cortex compression and buckling occur while the volar cortex remains intact [2,3]. These fractures differ from greenstick injuries, where the bone bends and bulges without a complete break, instead of being crushed, with one cortex being disrupted while the other is intact.

Traditionally, both clinicians and families had a widespread assumption that these fractures need a plaster cast immobilization for adequate healing [4]. Consequently, there is significant variation in how these fractures are treated across various hospitals and among different physicians. However, these fractures heal adequately well due to an intact periosteum, removable splints, and casts could be safe and effective alternatives to cast immobilization. Given the stable nature of this injury, recent literature has shown that alternative, more effective management options are available [4]. It has shown that even bandages are an adequate treatment [4]. For optimal pain relief, these fractures are typically best managed for a couple of weeks using a removable splint, soft cast, or soft bandage [5]. Routine follow-up is not necessary. Studies have shown that removing immobilization at home can further streamline management and has been shown to produce outcomes comparable to clinic-based re-examination and removal [6]. Since these fractures are generally self-limiting and rarely result in complications, current treatment strategies focus on reducing unnecessary interventions while promoting optimal functional recovery.

We conducted a retrospective audit in children younger than 16 years of age presenting with distal radius buckle fractures between August and October 2017. We compared our results with the National Institute for Health and Care Excellence (NICE) guidelines. We implemented a pathway in the ED and fracture clinic and then performed a second audit.

## Materials and methods

We conducted a retrospective study in the format of a closed-loop audit looking at the management of distal radius torus fractures in children at Queen’s Hospital, Burton Upon Trent, United Kingdom. We included all patients under the age of 16 years who presented to the ED with radiographic confirmation of a distal radius torus fracture. Patients 16 years of age and older, greensticks, and other hand injuries were excluded. Patient demographics, clinical letters, radiographs, and final treatment outcomes and cast application were collected and analyzed. Data was collected and analyzed using SPSS Statistics for Windows, Version 16 (Released 2007; SPSS Inc., Chicago, United States).

In the first audit, 65 children were included in the study between August 1, 2017 and October 30, 2017, which were reviewed in the ED and fracture clinic. In December 2017, the data were presented, and a pathway was developed and disseminated to both the fracture clinic and ED department with the aim of aligning our practices with the NICE guidelines. The NICE guidelines for managing distal radius buckle fractures in children recommend using removable splints instead of full casts to allow for quicker recovery, improved comfort, and reduced need for follow-up appointments. The information leaflets were not being used effectively. To resolve this, after presenting our initial findings at our departmental meeting, posters were displayed in the ED and fracture clinic. Additionally, email reminders were sent to all staff to ensure awareness of the NICE guidelines for treating these fractures. This resulted in the education of both the staff and parents about the fracture type and management.

A second audit was then performed on patients attending the fracture clinic between July 1, 2018 and September 30, 2018, which re-reviewed the practice in our unit after the introduction of the new pathway.

**Figure 1:**
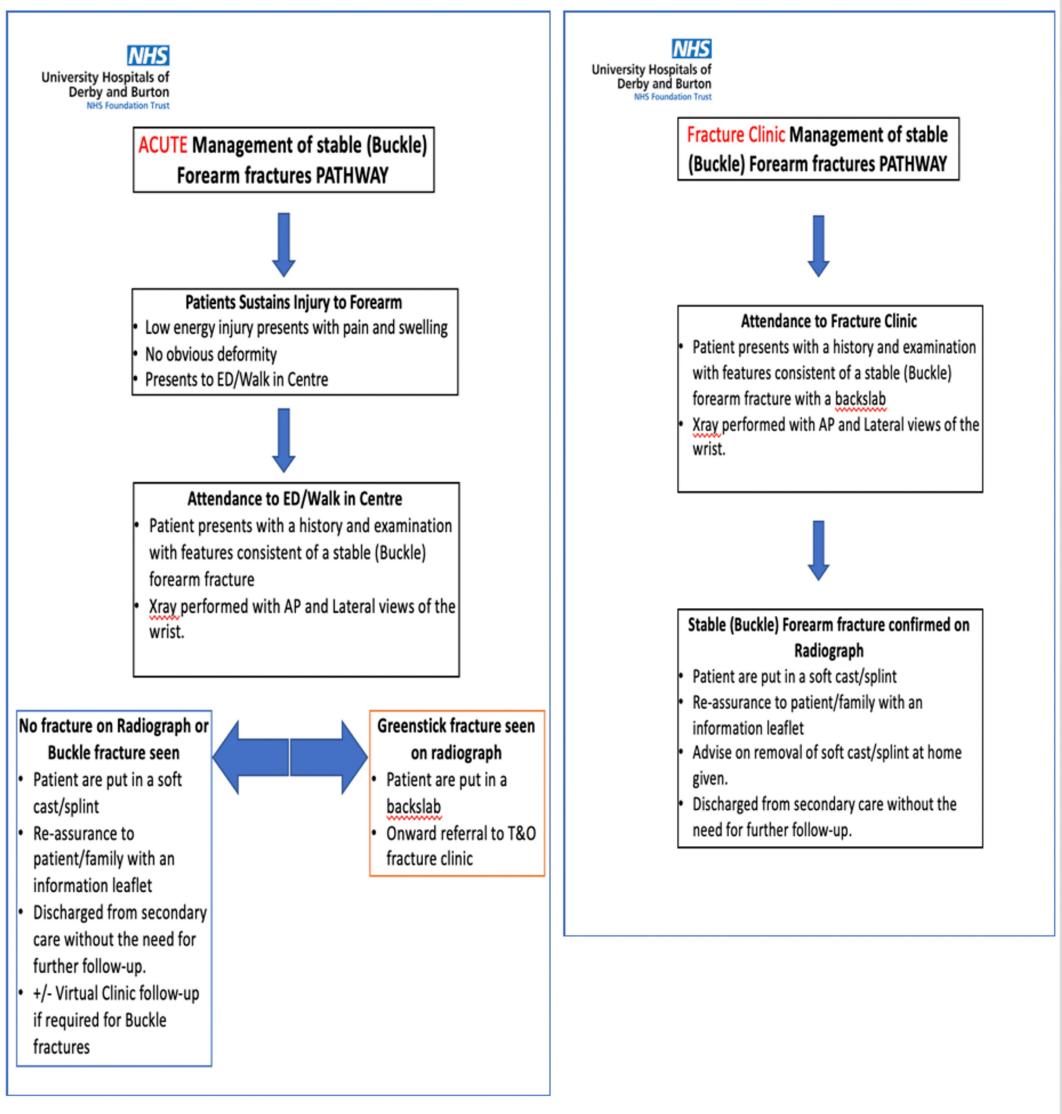
Pathways for management of stable (buckle) forearm fracture in emergency department (left) and fracture clinic (right) T&O: trauma and orthopedics

After the introduction of the new pathway in December 2017, patients attending ED were given a splint or soft cast by the ED personnel with an information leaflet to the parents or patient, with no further follow-up in the fracture clinic. The patients who were seen in the fracture clinic with a back slab were given a splint or soft cast, which can be removed easily at home with an information leaflet, and then discharged with no further follow-up (Figure 1).

## Results

A total of 152 cases with torus fractures were prospectively included in the two cycles, of which 65 and 87 children were included in audit cycles I and II, respectively. The demographics and mean age are presented in Table 1.

**Table 1:** Basic demographics for both audits

Basic demographics	
Total	152
Male (n)	96 (63.16%)
Female (n)	56 (36.4%)
Mean age (years)	8.5

First audit cycle

This included 65 patients; all patients were treated in a back slab in the ED. About 62 patients (95.4%) were treated in a full cast, with only three patients (4.6%) treated in a splint in a fracture clinic. Only five patients (7.7%) were discharged at the first visit, 56 patients (86.2%) attended a second visit, and a further four patients (6.2%) attended a third visit to the fracture clinic.

Second audit cycle

This included 87 patients, of which 70 (80.4%) were treated in a back slab, and 17 patients (19.5%) were treated in a splint in the ED. About 44 patients (50.6%) were converted to a splint, with 41 patients (47.1%) converted to a cast, and two patients (2.3%) remained in a splint in the fracture clinic. Around 39 patients (44.8%) were discharged on the first visit from the fracture clinic, and 48 patients (55.2%) attended the fracture clinic for a second visit.

Comparative results

**Figure 2:**
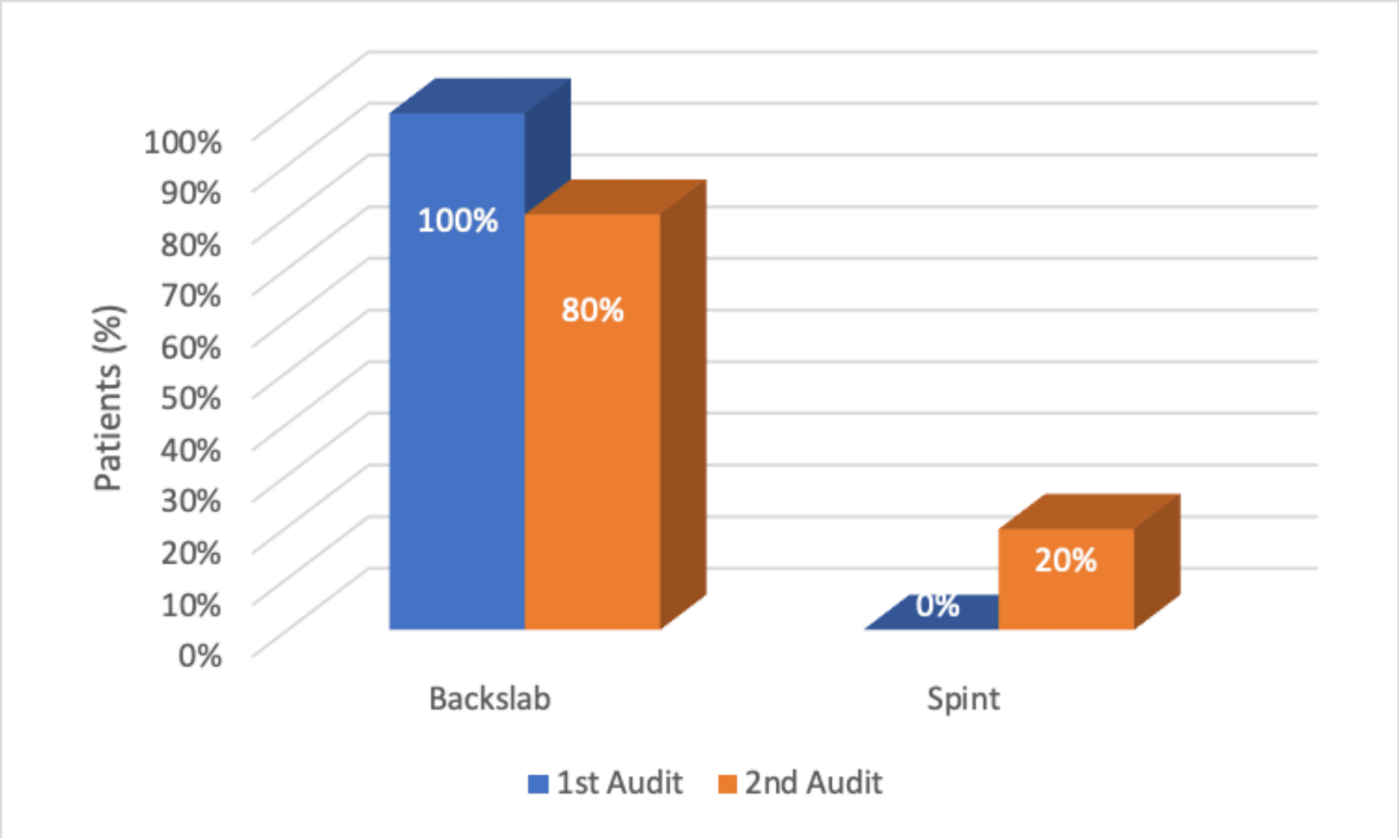
Initial treatment in the emergency department (ED)

**Figure 3:**
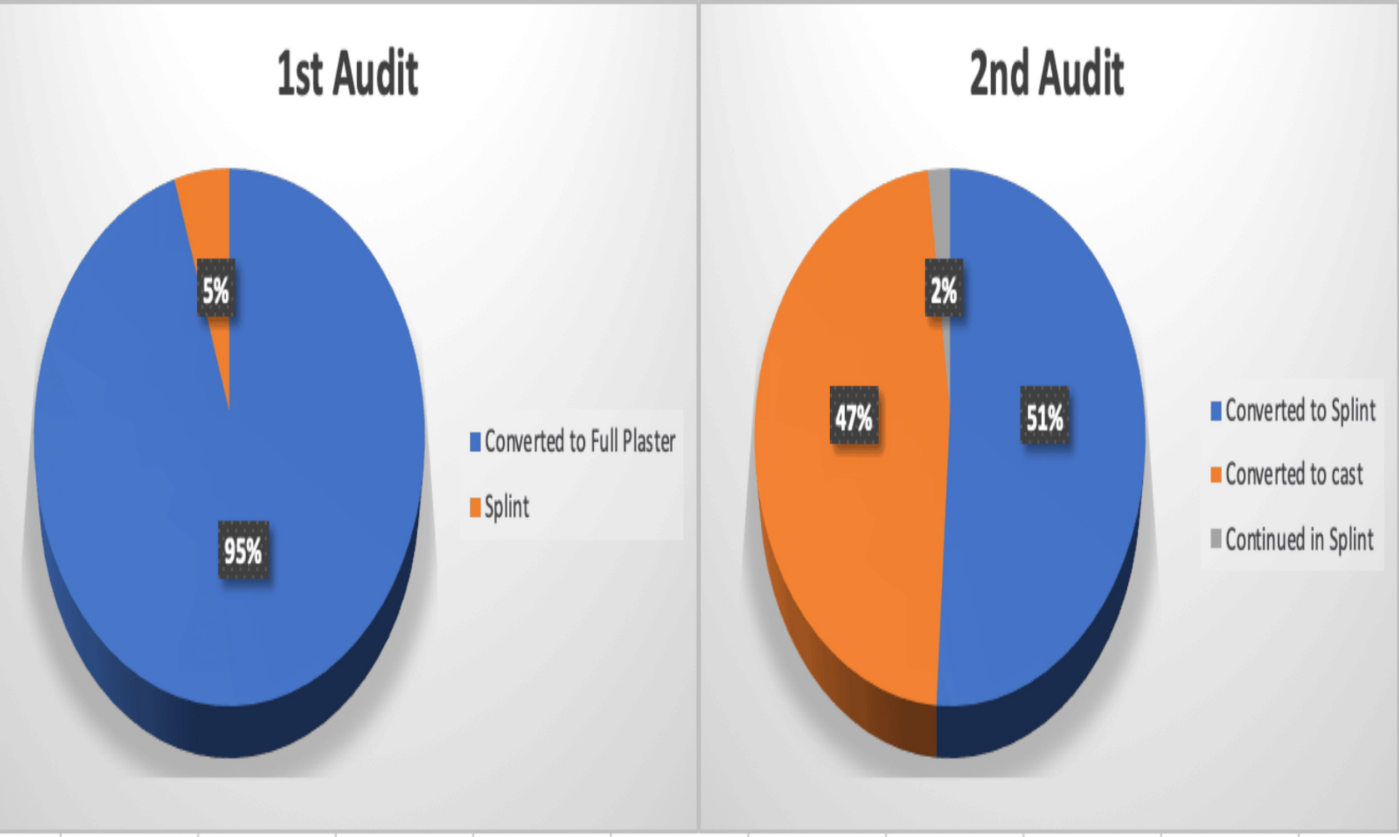
Treatment in fracture clinic (first audit versus second audit)

During the first cycle, 65 children (100%) were treated in a back slab in ED. Following our recommendations, 20% were treated with a splint and 80% with a back slab, as shown in Figure 2. In the fracture clinic, 95% of patients were treated with a full plaster, while only 5% were treated with a splint during the first cycle. In the second cycle, 53% were treated in a splint, and 47% were treated in a cast, as shown in Figure 3.

**Figure 4:**
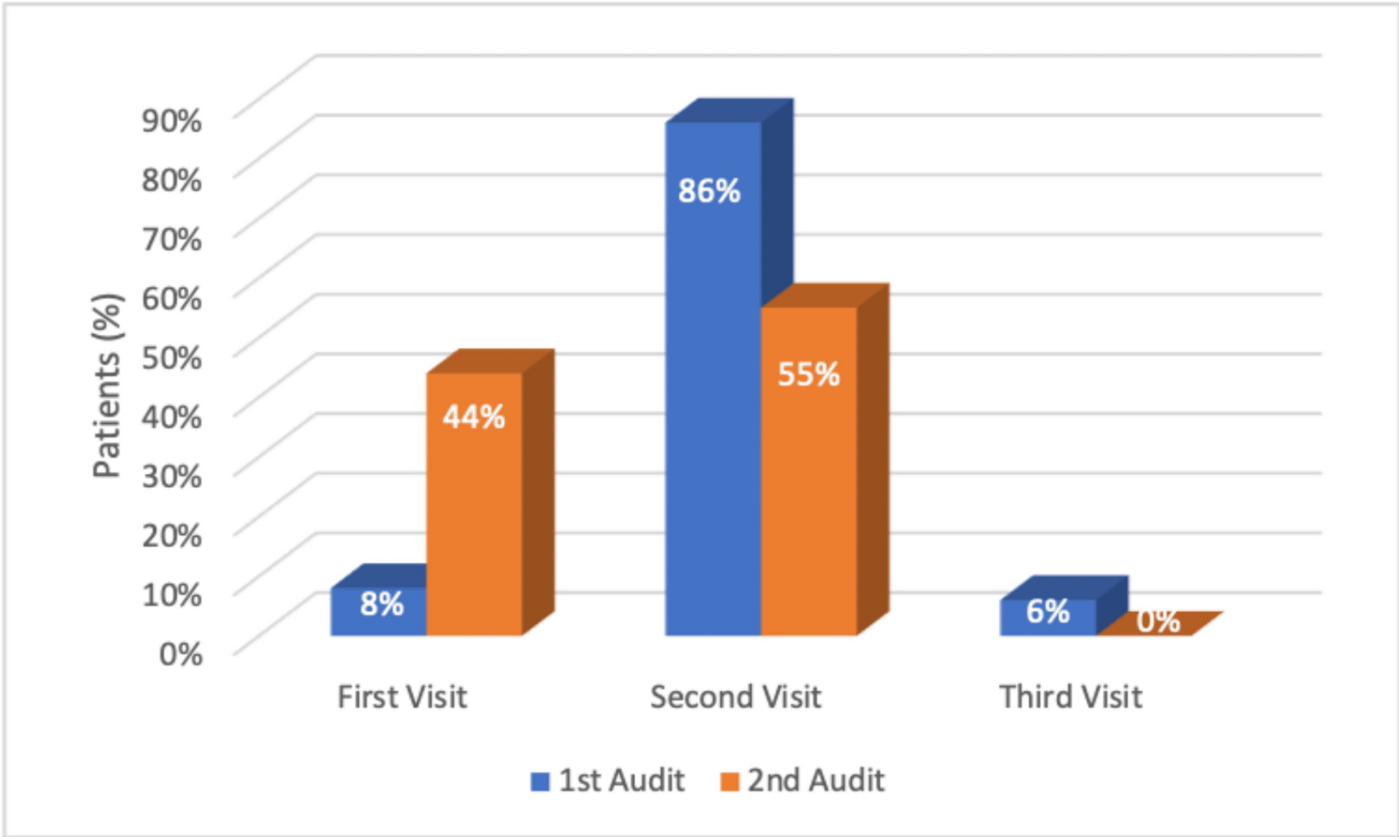
The number of fracture clinic visits

The number of patients discharged at clinic visits is shown in Figure 4. Out of the 65 children, 7.69% were discharged at the first visit, 86.2% at the second visit, and 5.15% at the third visit during the first cycle. In the second cycle, 44% of patients were discharged at the first visit and 55% during the second visit. One patient was on holiday through his follow-up period and was seen 45 days post-injury, so no treatment was given.

## Discussion

This audit illustrates how compliance with NICE guidelines for managing pediatric distal radius torus fractures has improved, although more must be done. The trend is shifting from rigid casting toward removable splints, reflecting recent evidence demonstrating equivalent outcomes with a less invasive approach [4]. Additionally, a recent study revealed that there were more cast-related complications requiring unscheduled clinic visits for cast change or removal, whereas no complications were observed in the splint group [7]. The use of splints for managing buckle fractures has been associated with high levels of patient satisfaction, primarily due to their comfort and ease of use. Unlike traditional casts, which can be bulky and limiting, splints allow for greater mobility and flexibility, enabling patients to engage in daily activities with minimal disruption [8]. Given these advantages, the shift toward splint use in the management of buckle fractures not only enhances clinical outcomes but also aligns with the increasing emphasis on patient-centered care.

The rate of discharge at the first visit remains suboptimal. Recent developments support the use of even less intervention than this. Hussain et al. (2024) summarize evidence to indicate that soft bandaging with immediate discharge represents optimal management [9]. This approach not only reduces healthcare utilization but also ensures favorable clinical outcomes, thus enhancing patient satisfaction and convenience [10]. As supported by NICE (2016) guidelines, the continued follow-up visits for many patients in our findings present the potential for further streamlining of care [1]. By implementing strategies that prioritize early discharge with appropriate home-care instructions, healthcare providers can optimize resource allocation while maintaining the quality of care.

Diagnosis is important for management to be appropriate. Tahir et al. (2024) state that clinicians must accurately differentiate torus fractures from other distal radius injuries to avoid overtreatment [11]. Radiographic feature education with clinical assessment may improve diagnostic accuracy. According to Williams et al. (2013), there are still some controversies on the optimal degree of immobilization [8]. The inconsistent practice and poor understanding of guidelines lead to unnecessary overcrowding in fracture clinics, prolonged waiting times, excessive radiograph use, and patient/caregiver inconvenience. As a result, these factors can increase healthcare spending and create additional financial burdens for caregivers due to added travel and time away from work [8].

While Perry et al. (2021) question whether any immobilization is required, our audit shows reluctance for the complete abandonment of supportive devices [4]. This is corroborated by the work of Baig and Egan (2017), who indicate that immobilization practices vary [12]. Further studies into patient/parent preference and long-term functional outcomes may inform practice evolution.

This study had several limitations. We performed a retrospective analysis of a small patient sample size, which may have influenced our results and outcomes. Additionally, the study did not consider the potential number of patients misdiagnosed during the initial assessment, which could have led to unnecessary management and follow-up. A recent paper has shown that, among trained radiographs, there has been a significant number of misdiagnoses of buckle fractures, which is an issue that is readily acknowledged [13]. Our study did not evaluate this issue, which may have influenced our results.

The lack of technician availability on weekends and the limited range of splint sizes may have negatively impacted the use of a back slab as an alternative, making it difficult to replicate in our results. Additional similar studies across multiple centers and multi-settings are needed to evaluate the treatment patterns for these stable injuries.

## Conclusions

Although our audit showed an improvement in adhering to the NICE guidelines for managing pediatric distal radius torus fractures, there is tremendous room for improvement in compliance. Education of all staff regarding best practices and easy availability of appropriate splints are key points for future improvement. These efforts aim to reduce unnecessary follow-up appointments by decreasing the burden of fractured clinic resources. This will ensure the optimization of care pathways for this common pediatric injury and an overall advantage to patients and healthcare systems in promoting efficiency and evidence-based management.
